# Case Report of a Complex Colovesical Fistula Associated With an Infected Artificial Urinary Sphincter: Radiological and Clinical Manifestations and Treatment Discussion

**DOI:** 10.7759/cureus.116314

**Published:** 2026-09-15

**Authors:** Johan Harris, Jack Gurwin, David Staskin, Nour Nakrour, Rania Hamada, Alaa Hamada

**Affiliations:** 1 Urology, Wayne State University, Detroit, USA; 2 Urology, Wayne State University School of Medicine, Detroit, USA; 3 Boston Medical Center, Tufts University School of Medicine, Boston, USA; 4 Radiology, John D. Dingell VA Medical Center, Detroit, USA; 5 Surgery, Wayne State University, Detroit, USA; 6 Surgery/Urology, John D. Dingell VA Medical Center, Detroit, USA

**Keywords:** artificial urinary sphincter, colo vesical fistulae, cuff erosion, entero-colonic fistula, infection

## Abstract

Surgical placement of an artificial urinary sphincter (AUS) is considered the gold standard treatment for stress urinary incontinence. However, this surgery is not without complications such as infection or AUS cuff erosion. In this case report, we describe delayed AUS cuff infection/cuff erosion (seven months post-placement) concomitantly seen in an 82-year-old man with colovesical and colocolonic fistulas. In the past, this patient with severe post-prostatectomy mixed urinary incontinence had undergone multiple previous continence operations, including prior repeated AUS placement, Bulkamid injection (Boston Scientific Corporation, Marlborough, MA), ProACT insertion (Uromedica, Inc., Plymouth, MN), and Botox bladder injection. This presentation led to a life-threatening infection and required immediate complex surgical intervention. We will discuss clinical and radiological diagnostic features and will highlight the potential risk factors for this delayed erosion.

## Introduction

Artificial urinary sphincter (AUS) is currently the gold standard treatment option for male stress urinary incontinence. Despite its long-term success, AUS placement has intraoperative and postoperative complications, including infection, erosion, bleeding, and reoperation. Infection of AUS was reported around 0-14% and is divided into early (<4 months) and late (>4 months) [1-4]. Early infected AUS is a possible concomitant event with AUS urethral erosion or intraoperative contamination. The most common cultured pathogens are skin flora. Late infections are thought to be the result of hematogenous seeding secondary to bacteremia from other sources [5].

Erosion of the AUS cuff into the urethra is generally classified as either early or late postoperatively; however, no specific time threshold has been established to differentiate between the two categories. Whereas early erosion can be related to technical errors, late erosion is attributed to prolonged catheterization or catheterization without deactivation of the device. Radiation, advanced age, and fragile urethra due to prior erosions or urethroplasty have been reported as risk factors [6-11]. Infection of the AUS frequently occurs concomitantly with AUS erosion. A recent pooled analysis reported a urethral erosion rate of approximately 8.5% following AUS placement, with reported rates ranging from 3.3% to 27.8% [12].

In this case report, delayed infection and AUS urethral cuff erosion presented in a patient who had concomitantly developed colovesical and colocolonic fistulas and presented with necrotizing fasciitis, a large perineal abscess, and septicemia. The first objective of presenting this case report is to highlight the radiological and clinical manifestations of this patient with delayed AUS cuff erosion/infection who had a possibly compromised urethra from previous continence surgeries and had a concomitant complex colovesical fistula. The second objective is to discuss treatment options for patients with severe post-prostatectomy mixed urinary incontinence and a compromised urethra who have failed multiple operations.

## Case presentation

The case report describes an 82-year-old White American male patient with a longstanding history of severe mixed incontinence, using 6-7 pads daily post-radical prostatectomy in 2003. The patient denied previous radiation. The patient had poor response to mirabegron, oxybutynin, and trospium. A urodynamic study (UDS) showed bladder capacity of 499 cc, good compliance, intact sensation, and presence of detrusor overactivity (DO) (see Figure 1).

**Figure 1:**
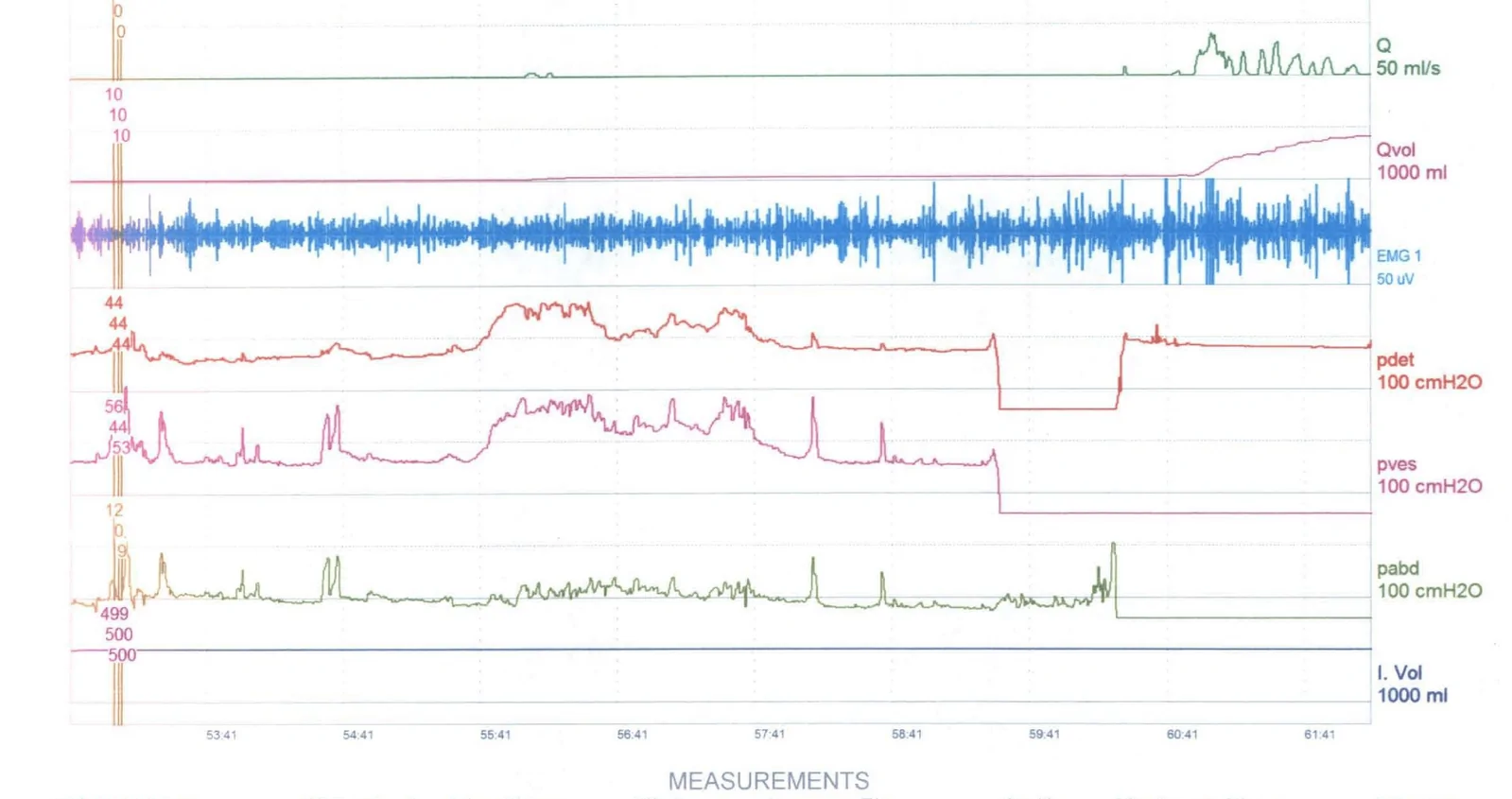
Urodynamic study (UDS) of the bladder demonstrating detrusor overactivity (DO).

He underwent his first placement of AUS in 2018 with a 4.5 cm cuff size and 61-70 PRB with 23 cc saline. However, he experienced worsening of his overactive bladder (OAB) with worsening urge incontinence, and he used more than 10 pads per day. Flexible cystoscopy revealed good coaptation and intact device cycling with no erosion. He had been undergoing Botox injections every three months with partial response; however, he eventually failed intravesical Botox injections as well.

In 2022, he underwent temporary, followed by permanent placement of an InterStim sacral neuromodulator (Medtronic, Minneapolis, MN). Despite initial response, his irritative lower urinary tract symptoms (LUTS) and urge incontinence worsened despite increasing the stimulation amplitude to 3.2 mA.

Three months later, in May 2022, it was determined that AUS revision was indicated, with placement of a new 4 cm cuff at a more distal bulbar urethral location. The device was deactivated for three months as there was suspected urethrocutaneous fistula and urine leak via the perineal incision. This was managed with an indwelling Foley catheter. Flexible cystoscopy was then performed, which showed no urethral erosion, and the device was cycled successfully. The patient, however, was unable to cycle the pump and had urine retention, and the device was deactivated. The following month, the device was reactivated, and the patient began to use it, but his incontinence worsened, and he required 10 diapers/day. The patient then had a perineal abscess, which was subsequently drained. The patient was advised about explantation of the device, and this was performed in January 2023.

Three months later, he underwent cystoscopy with Botox injection with limited benefit, followed three months later by combined Botox and Bulkamid injection (Boston Scientific Corporation, Marlborough, MA). This resulted in approximately 50% improvement. Six months later, in February 2024, he underwent repeat bladder Botox and Bulkamid injections; however, his incontinence subsequently worsened, with the patient requiring 10 pads per day. A urodynamic study was then performed, demonstrating DO with uncontrolled urine leakage. The patient was then counseled about the ProACT device (Uromedica, Inc., Plymouth, MN), which was inserted in October 2024. However, two revisions were performed because of malposition, and eventually the device eroded through the scrotal skin. The device was eventually removed in February 2025.

With frustration about his incontinence and the need for 16 pads per day, the patient underwent another workup with UDS and cystoscopy, which showed no evidence of stricture. UDS demonstrated an adequate bladder capacity of 400cc with normal compliance and DO, with no demonstrable leak on Valsalva/cough. The patient then underwent placement of a new AUS in July 2025. Intraoperatively, the urethral wall was thin, and a 4.5 cm cuff was placed using a transcorporal approach. The patient, however, continued to complain about persistent leakage requiring approximately 10 pads per day. A repeat cystoscopy was performed in January 2026, which demonstrated no evidence of AUS urethral cuff erosion and good cycling of the device; however, a scarred urethra was noticed. In February 2026, the patient presented to the emergency department at John D. Dingell Veterans Affairs Hospital in Detroit, Michigan, with fever, chills, and scrotal and pelvic pain. Subsequent CT scan showed colovesical and colocolonic fistulas, air in the bladder, fluid with gas formation around the AUS cuff, urethral erosion, and a perineal abscess (Figures 2-4).

**Figure 2:**
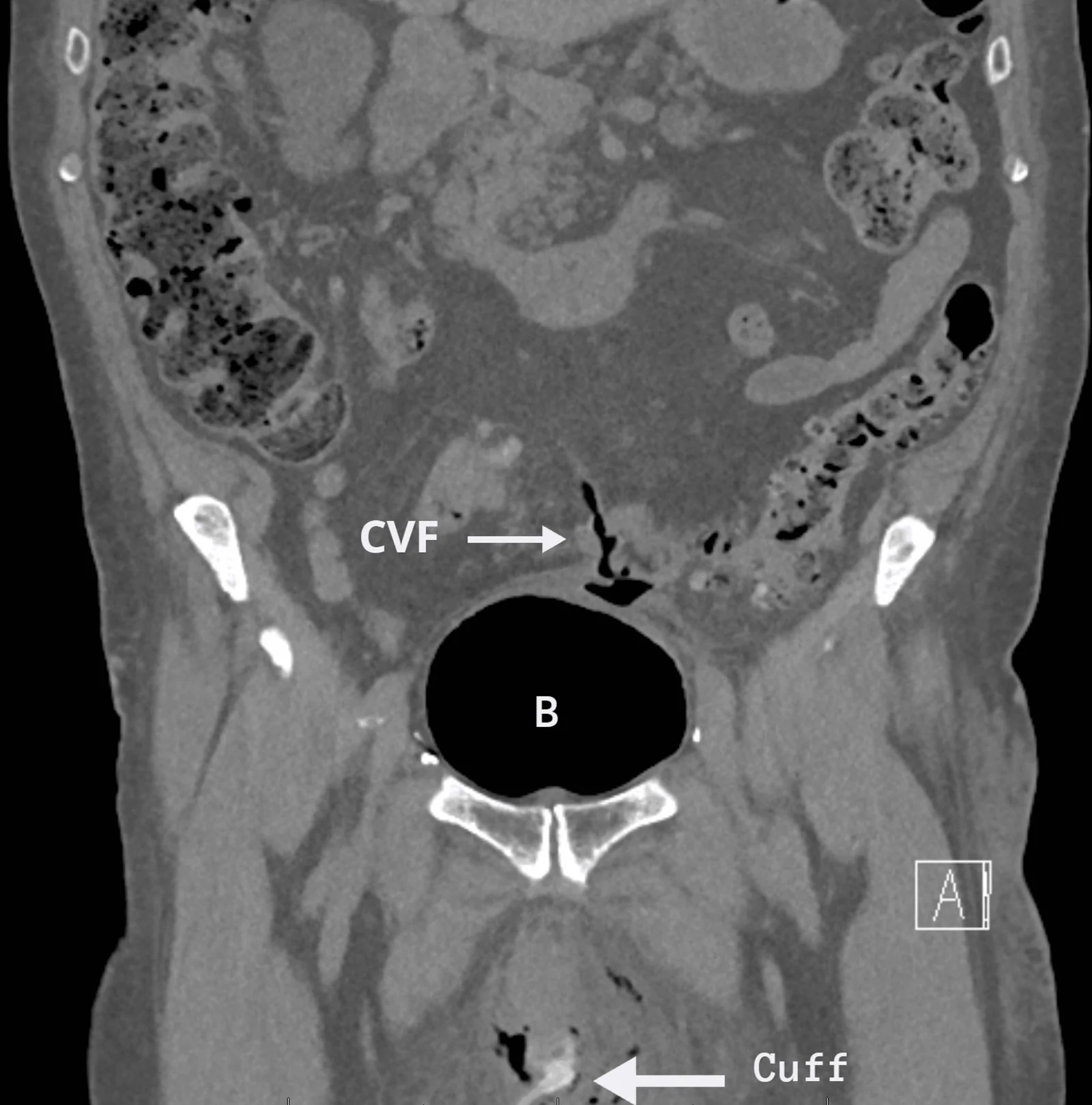
CT abdomen and pelvis (coronal view) showing air within the bladder (B), colovesical fistula (CVF) and air around the AUS cuff (Cuff).

**Figure 3:**
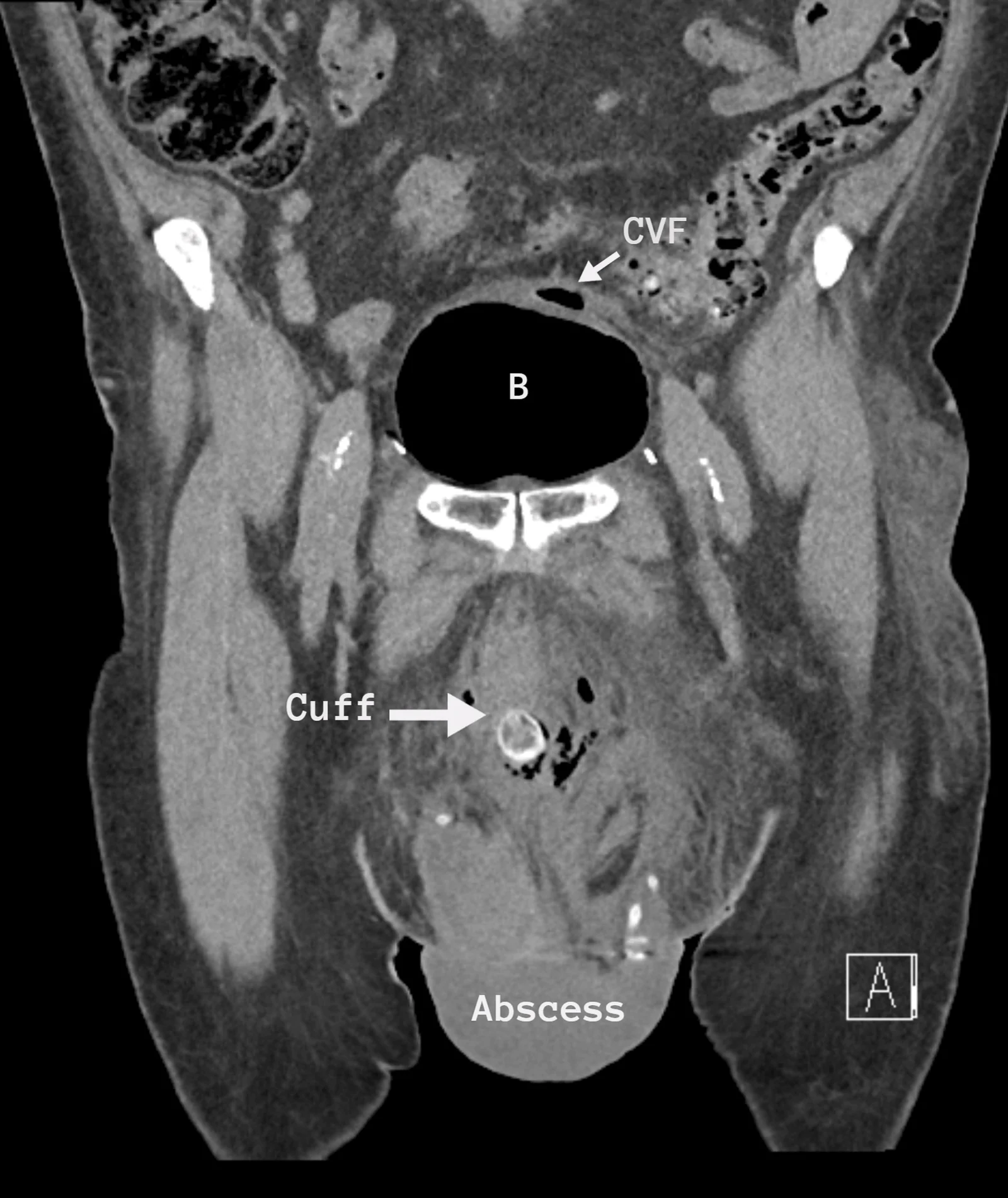
CT abdomen and pelvis (coronal view) showing air in the bladder (B), colovesical fistula (CVF), AUS cuff (Cuff) with erosion into the urethra and perineal abscess (Abscess).

**Figure 4:**
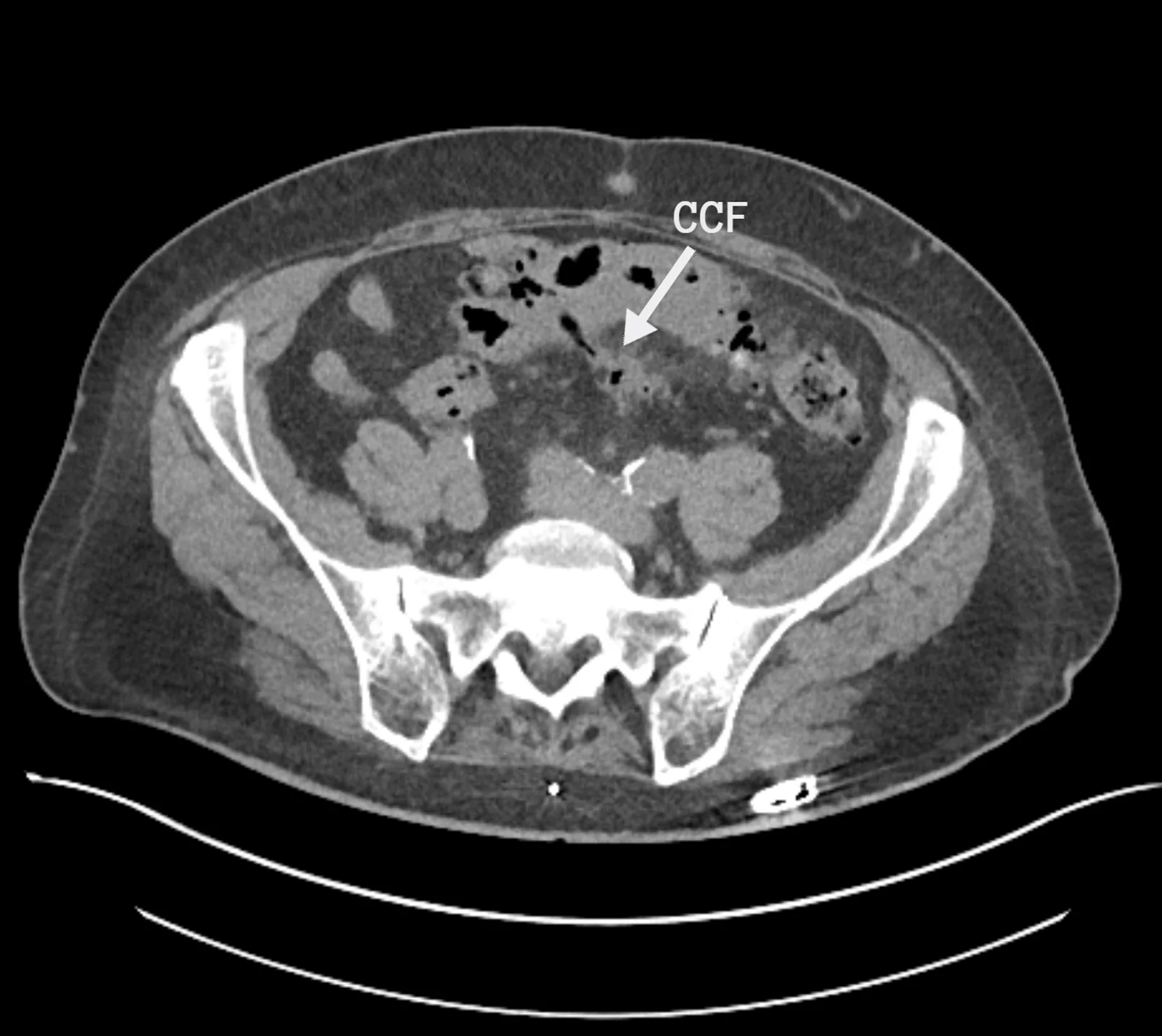
CT abdomen and pelvis (axial view) showing colocolonic fistula (CCF).

Emergency perineal exploration was performed, and copious foul-smelling pus was drained. After the cuff was removed, the urethra was apparently eroded; repair was done using interrupted 4-0 Vicryl, and a Foley catheter was placed. Open suprapubic cystostomy was also performed through a lower midline incision for maximal urinary diversion. General Surgery was consulted and then performed a loop colostomy for fecal diversion. The perineal incision was kept open to heal by secondary intention using a wound VAC, and within six weeks, with adequate wound care, his incision healed nicely. A retrograde urethrogram was performed at six weeks, which showed no extravasation, and the penile Foley catheter was removed. The patient was advised to continue to exchange the suprapubic tube indefinitely.

## Discussion

To our knowledge, this is the first case reporting combined delayed urethral erosion by the cuff of an artificial urinary sphincter with severe necrotizing infection and concomitant colovesical and colocolonic fistulas. Early (<4 months) AUS infection is typically rare in the absence of concomitant erosion, which can be accidentally missed intraoperatively. Late AUS infection, however, may be related to delayed erosion from improper catheterization, prior radiation, or hematogenous spread. Cystoscopy is the key to confirming the presence of urethral erosion. Additional risk factors reported for AUS cuff erosion that may be relevant to our patient include age >80 years, frailty, and prior AUS erosion.

It was reported that placing gastrointestinal segments in contact with the urinary system, such as in augmentation cystoplasty, has been associated with a higher incidence of early AUS infection/erosion in cases of concomitant placement. This can be related to intraoperative and postoperative bacterial contamination despite preoperative effort to sterilize the bowel prior to combined surgery. Holmes et al. reported a 43% (3/7) AUS erosion rate among patients undergoing simultaneous gastrocystoplasty and AUS implantation, with a mean time to erosion of two years [13]. In contrast, Delgado-Miguel et al. found no difference in AUS erosion rates between concomitant placement (3/27 patients) and staged procedures (2/12 patients) [14]. Despite this finding, the overall erosion rate in this small sample study group remained relatively high at 13% (5/39 patients). Suggested etiologies included bacteriuria, inappropriate cuff size, tissue ischemia, and atrophy.

Rare cases of bowel erosion associated with AUS components have been reported in the literature. Yuan et al. reported incidental discovery of small bowel erosion by the AUS reservoir during lysis of peritoneal adhesion during sacrocolpopexy and cystolithotomy in a patient without significant urinary symptoms apart from bladder calculus [15]. In that case, salvage replacement of the AUS reservoir and tubing was performed with an uneventful outcome. In contrast, Shieh et al. described sigmoid colon erosion by the AUS tubing in a patient with a sliding hernia and concomitant AUS infection [16]. Intraoperatively, the AUS tubing was eroding into the sigmoid colon inside the hernia, necessitating AUS component removal and hernia and sigmoid colon repair.

By reviewing UDS prior to his most recent AUS placement, it was determined that the patient had documented DO with no demonstrable leak on Valsalva maneuver/cough; however, there was demonstrated leakage on positional change, with leakage onto the floor. The patient also complained of persistent leakage for seven months post AUS placement. It has been reported that OAB symptoms may improve in 60% of patients with mixed incontinence post AUS placement [17,18]. Bladder capacity <200 cc and prior radiation were risk factors for persistent OAB symptoms [17,18]. Our patient has adequate bladder capacity of 400cc and no prior radiation exposure. It is uncertain, though, with this protracted history, mixed incontinence, UDS findings, and persistent leakage post-placement, whether AUS placement was a reasonable decision. 

During his most recent AUS placement surgery, transcorporal (TC) cuff placement was selected because of the thin urethral wall per the operative report. In a recent systematic review of eight comparative studies, Gutiérrez et al. showed that TC AUS was associated with significantly lower infection (OR 0.33, 95% CI 0.12-0.95) and erosion rates (OR 0.35, 95% CI 0.15-0.81) [19]. Subgroup analysis in the high-risk urethra population demonstrated similar trends. Pandit et al. suggested some operative treatment options for fragile urethra, such as TC cuff placement, urethral wrapping, and use of a lower-pressure regulating balloon [20]. However, none of these options have high-quality evidence, and their outcomes are not always reproducible. Pandit et al. suggested other alternatives such as pad use, suprapubic tubes, urethral ligation, or simple cystectomy with diversion.

It is recommended that treatment of delayed erosion in the absence of infection is surgical explant. However, Shumaker et al. reported five cases of delayed (placed > 1 year) AUS cuff urethral erosion treated with either delayed removal or no explant [21]. Given the presence of concomitant necrotizing subcutaneous infection, abscess with gas formation, and sepsis, our patient required immediate colostomy, explantation of AUS components, and suprapubic cystostomy as life-saving interventions.

## Conclusions

Delayed urethral cuff erosion/infection due to a fragile urethra and severe UTI due to a colovesical fistula was a highly likely outcome despite transcorporal placement of the cuff. Proper patient selection to replace AUS is highly recommended. Clinicians dealing with patients with severe mixed incontinence should consider the severity of concomitant OAB before offering AUS placement, as it can worsen surgical outcomes. In addition, prior failure of multiple AUS devices must be a strong warning for possible complications of pursuing additional implants. A fragile urethra constitutes a challenge for successful AUS placement, as delayed erosion is a potential complication despite surgical modifications to minimize erosion risk. Urine diversion may offer a minimally invasive option in patients with mixed incontinence and a demonstrated refractory OAB component. 
